# Validity and Reliability of the Inertial Measurement Unit for Barbell Velocity Assessments: A Systematic Review

**DOI:** 10.3390/s21072511

**Published:** 2021-04-03

**Authors:** Filipe Manuel Clemente, Zeki Akyildiz, José Pino-Ortega, Markel Rico-González

**Affiliations:** 1 Instituto Politécnico de Viana do Castelo, Escola Superior Desporto e Lazer, Rua Escola Industrial e Comercial de Nun’Álvares, 4900-347 Viana do Castelo, Portugal; 2 Instituto de Telecomunicações, Delegação da Covilhã, 1049-001 Lisboa, Portugal; 3 Sports Science Department, Gazi University, Teknikokullar, Ankara 06500, Turkey; zekiakyldz@hotmail.com; 4 Faculty of Sports Sciences, University of Murcia, San Javier, 30100 Murcia, Spain; josepinoortega@um.es; 5 BIOVETMED & SPORTSCI Research Group, Department of Physical Activity and Sport, Faculty of Sport Sciences, University of Murcia, San Javier, 30100 Murcia, Spain; markeluniv@gmail.com; 6 Department of Physical Education and Sport, University of the Basque Country, UPV-EHU, Lasarte 71, 01007 Vitoria-Gasteiz, Spain

**Keywords:** sports technology, sensors, accuracy, precision, performance, velocity-based training

## Abstract

The use of inertial measurement unit (IMU) has become popular in sports assessment. In the case of velocity-based training (VBT), there is a need to measure barbell velocity in each repetition. The use of IMUs may make the monitoring process easier; however, its validity and reliability should be established. Thus, this systematic review aimed to (1) identify and summarize studies that have examined the validity of wearable wireless IMUs for measuring barbell velocity and (2) identify and summarize studies that have examined the reliability of IMUs for measuring barbell velocity. A systematic review of Cochrane Library, EBSCO, PubMed, Scielo, Scopus, SPORTDiscus, and Web of Science databases was performed according to the Preferred Reporting Items for Systematic Reviews and Meta-Analyses (PRISMA) guidelines. From the 161 studies initially identified, 22 were fully reviewed, and their outcome measures were extracted and analyzed. Among the eight different IMU models, seven can be considered valid and reliable for measuring barbell velocity. The great majority of IMUs used for measuring barbell velocity in linear trajectories are valid and reliable, and thus can be used by coaches for external load monitoring.

## 1. Introduction

Velocity-based training (VBT) is a resistance training method consisting of monitoring the velocity of movement displacement to support the regulation of load imposed on athletes [1,2,3,4,5]. Therefore, a proper measurement of bar displacement velocity is critical to implement an auto-regulation process of sports training [6,7,8,9,10]. Three reasons can be cited for using velocity as the main outcome [11,12,13]. First, there is a relationship between velocity and the amount of external mass lifted, by which a reduction in lifting velocity occurs as load increases until a terminal velocity is achieved at the maximal load [14]. Second, a nearly perfect linear relationship between velocity and intensity can be observed in many exercises and movements performed at different loads [15,16,17]. Third, reductions in voluntary exercise velocity are strictly related to neuromuscular fatigue induced by the exercise [18,19,20].

If an athlete is to benefit from VBT, certain instruments should be used to ensure that the velocity of movements is accurately and precisely measured [21,22]. For this purpose, different commercial devices can be used to quantify velocity [23]. Among the available options, solutions can be grouped as follows [24]: (i) isoinertial dynamometers consisting of a cable-extension linear velocity transducer attached to the barbell [25,26,27], (ii) optical motion sensing systems or optoelectronic systems [28,29,30,31], (iii) smartphone applications involving frame-by-frame manual inspections [29,32,33], and (iv) inertial measurement units (IMUs) [34]. Since these different technologies offer different possibilities, it can be considered that IMUs represent the most easy-to-use solution because no cable-extension is needed—the sensor simply needs to be attached to the barbell. Compared with video-based solutions, IMUs are also easier and quicker since no operations need to be made [35,36].

IMU solutions use fusion sensing to estimate velocity [37]. Thus, despite their practical benefits, some issues related to accuracy and precision should be considered. IMUs combine accelerometers (usually triaxial), a gyroscope (usually triaxial), and magnetic sensors to provide information about velocity, orientation, and gravitational force [35,38]. Despite the combination of sensors, there is always a margin of error related to the accuracy and precision of the estimations [39]. This margin of error should be understood so that better inferences can be made about human performance variability [40]. In fact, if validity or reliability is neglected, the results can be misunderstood, possibly affecting the judgments of coaches about their athletes [41,42,43,44,45].

On the basis of the importance of confirming the validity and reliability of IMU devices, different original studies have reported the results for different models in the sports sciences community [46,47,48,49]. Naturally, different experimental protocols have led to different results, and not all of the models are covered in the same conditions. Therefore, there is a need for a systematic review summarizing the validity and reliability levels of different IMU models during barbell movements. This will help us to understand whether coaches and athletes can use this technology to monitor resistance training that considers variations in human performance as opposed to in the devices [50].

While several systematic reviews have been published about the use of IMUs [50,51,52,53], no systematic review has summarized the validity and reliability levels of different IMU models for measuring barbell velocity. Considering the importance of the accuracy and precision level of determining barbell velocity in providing adequate prescriptions of resistance training, the aim of the present systematic review was twofold: (1) to identify and summarize studies that have examined the validity of wearable wireless IMU for measuring barbell velocity, and (2) to identify and summarize studies that have examined the reliability of IMUs for measuring barbell velocity.

## 2. Materials and Methods

The systematic review strategy was conducted according to PRISMA (Preferred Reporting Items for Systematic Reviews and Meta-Analyses) guidelines [54]. The protocol was registered with the International Platform of Registered Systematic Review and Meta-Analysis Protocols with the number 2020120135 and the DOI number 10.37766/inplasy2020.12.0135.

### 2.1. Eligibility Criteria

The inclusion and exclusion criteria can be found in Table 1.

The screening of the title, abstract, and reference list of each study to locate potentially relevant studies was independently performed by 2 of the authors (F.M.C. and M.R.G.). Additionally, they reviewed the full version of the included papers in detail to identify articles that met the selection criteria. An additional search within the reference lists of the included records was conducted to retrieve additional relevant studies. A discussion was made in the cases of discrepancies regarding the selection process with a third author (Z.A.). Possible errata for the included articles were considered.

### 2.2. Information Sources and Search

Electronic databases (Cochrane Library, EBSCO, PubMed, SPORTDiscus, and Web of Science) were searched for relevant publications prior to 1 January 2021. Keywords and synonyms were entered in various combinations in the title, abstract, or keywords: (sport* OR exercise* OR “physical activit*” OR movement*) AND (“inertial measurement unit” OR IMU OR acceleromet* OR “inertial sensor” OR wearable OR MEMS OR magnetometer) AND (Validity OR Accuracy OR Reliability OR Precision OR Varia* OR Repeatability OR Reproducibility OR Consistency OR noise) AND (barbell OR bar). Additionally, the reference lists of the studies retrieved were manually searched to identify potentially eligible studies not captured by the electronic searches. Finally, an external expert was contacted in order to verify the final list of references included in this scoping review in order to understand if there was any study that was not detected through our research. Possible errata were searched for each included study.

### 2.3. Data Extraction

A specific Excel spreadsheet was prepared for data extraction (Microsoft Corporation, Readmon, WA, USA) following the guidelines of Cochrane Consumers and Communication Review Groups [55]. The spreadsheet was used to identify the accomplishment of inclusion or exclusion criteria and to support the selection of the articles. The process was made by 2 of the authors (F.M.C. and M.R.G.) in an independent way. After that, they compared the results, and any disagreement regarding the eligibility was discussed until a decision was made in agreement.

### 2.4. Data Items

The following information was extracted from the included original articles: (i) validity measure (e.g., typical error, absolute mean error) and (ii) reliability measure (e.g., intraclass correlation coefficient (ICC) and/or typical error of measurement (TEM) (%) and/or coefficient of variation (CV) (%) and/or standard error of measurement (SEM)). Additionally, the following data items were extracted: (i) type of study design, number of participants (n), age-group (youth, adults, or both), sex (men, women, or both), training level (untrained, trained); (ii) characteristics of the wearable wireless IMU and comparator (isoinertial dynamometer consisting in cable-extension linear position transducer or optoelectronic system); (iii) characteristics of the experimental approach to the problem, procedures, and settings of each study.

### 2.5. Methodological Assessment

The STROBE assessment was applied by 2 of the authors (J.P.O. and M.R.G.) to assess the methodological bias of eligible articles following the adaptation of O’Reilly et al. [51]. Each of the included articles was scored for 10 items [51]. In cases of disagreement, it was discussed and solved by consensus decision. The assessment process was made in an independent way. After that, both authors compared the results, and any disagreement regarding the scores were discussed and made a decision in agreement. The study rating was qualitatively interpreted following O’Reilly et al. [51]—from 0 to 7 scores, the study was considered as risk of bias (low quality), whereas, if the study was rated from 7 to 10 points, it was considered as a low risk of bias (high quality).

## 3. Results

### 3.1. Study Identification and Selection

**Figure 1:**
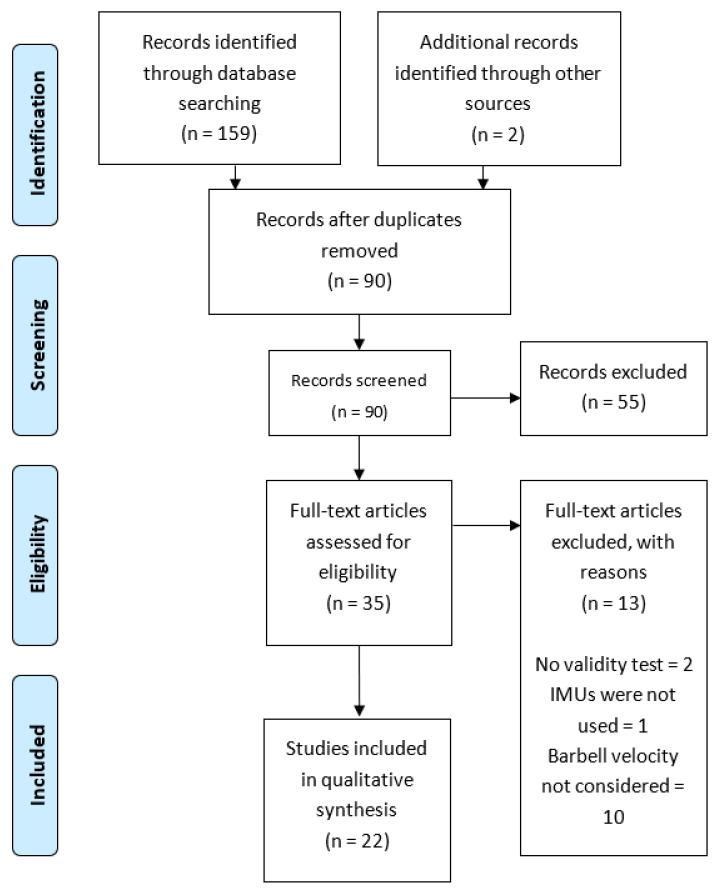
Preferred Reporting Items for Systematic Reviews and Meta-Analyses (PRISMA) flow diagram.

The searching of databases identified a total of 159 titles (Cochrane Library = 11; EBSCO = 59; PubMed = 31; SPORTDiscus = 31; Web of Science = 27). These studies, together with another two included from external sources, were then exported to reference manager software (EndNote X9, Clarivate Analytics, Philadelphia, PA, USA). Duplicates (71 references) were subsequently removed either automatically or manually. The remaining 90 articles were screened for their relevance on the basis of titles and abstracts, resulting in the removal of a further 55 studies. Following the screening procedure, 35 articles were selected for in-depth reading and analysis. After reading full texts, a further 13 studies were excluded due to not meeting the eligibility criteria (Figure 1).

### 3.2. Methodological Quality

The overall methodological quality of the cross-sectional studies can be found in Table 2.

### 3.3. Characteristics of Individual Studies

Characteristics of the included studies can be found in Table 3. Twenty-one of the included articles tested validity of the IMU [23,24,27,34,46,48,49,56,58,59,60,61,62,63,64,65,66,67,68,69,70]. Nineteen of the included articles tested the reliability of the IMU [23,24,27,34,46,48,49,56,57,58,60,61,62,64,65,66,67,68,69]. Six of the included articles compared the IMU with linear transducers [23,27,32,48,62,68]. Five of the articles compared the IMU with contact platform [56,58,59,60,67], while one of the articles [69] compared the IMU with field computation method. Finally, six of the articles compared the IMU with motion capture system [24,61,64,65,66,70].

Among the included studies, 10 tested the back squat [23,34,46,56,57,58,62,63,65,68]; 9 the bench press [1,23,24,34,49,58,64,66,69], 1 the hip thrust [34]; 1 the bench throw [58], 1 the prone bench pull [23]; 2 the counter movement jump [60,67]; 1 the power snatch, clean, jerk [61]; and 1 the hexagonal barbell deadlift [48].

Overall, eight different IMU models were tested, in which two studies were conducted using the Sensei Bar [27,46], one using the Gyko [49], six using the Myotest [56,58,59,65,67,69], six using the Push Band [23,24,48,54,64,66], four using the Wimu Real Track [6,60,62,68], two using the Pasco [61,70], and one using the Rehagait [63].

### 3.4. Results of Individual Studies: Validity of IMU for Estimation of Barbell Velocity

Information of the validity levels obtained in the included studies can be found in Table 4. Some of the studies listed in Table 4 were reported to be not valid (*n* = 4). Other studies were reported to be valid (n = 18). For the Barsensei model, the SEE values of validity were between 0.03 and 0.06 m•s^−1^ [46]. For the Gyko Sport model, the SEE values and Pearson’s *r* were 0.18 m•s^−1^ and *r* = 0.79, respectively [49]. For the Beast Sensor model, the SEE values were between 0.07 m•s^−1^ and 0.05 m•s^−1^ and Pearson’s *r* values were between 0.76 and 0.98 [24,34]. For the Myotest sensor model, the SEE values were between 0.01 m•s^−1^ and 26.6 m•s^−1^ and Pearson’s *r* values were between 0.38 and 0.92, and *R^2^* values were between 0.59 and 0.97 [56,58,59,65,67,69]. For the PUSH Band sensor model, the SEE values were between 0.135 m•s^−1^ and 0.091 m•s^−1^ and Pearson’s *r* values were between 0.97 and 0.90, and *R^2^* value was 0.85 [6,23,24,48,64,66]. For the Wimu RealTrack Systems sensor model, the SEE value was 0.030 m•s^−1^ and Pearson’s *r* values were between 0.009 and 0.60, and *R^2^* values were between 0.95 and 0.77 [28,60,68]. For the PASCO sensor model, Pearson’s *r* values were between 0.84 and 0.93 [61,70].

### 3.5. Results of Individual Studies: Reliability of IMU for Estimation of Barbell Velocity

Information of the reliability levels obtained in the included studies can be found in Table 5. Generally, the Barsensei model was the only model not being considered reliable for more than one article [27,46]. The remaining models presented evidence of reliability.

## 4. Discussion

This systematic review aimed to identify and summarize studies that have examined the validity of wearable wireless IMUs for measuring barbell velocity and identify and summarize studies that have examined the reliability of IMUs for measuring barbell velocity. The IMUs in this study were compared with gold standards and previously tested devices as reference systems (i.e., linear transducers [56,58,59,60,67], a contact platform [69], the field computation method [24,61,64,65,66,70], and a motion capture system).

IMUs were evaluated during movements generally geared toward strength training. The studies investigated in this review included the following movements: the back squat [1,23,24,34,49,58,64,66,69]; the bench press [34]; the hip thrust [58]; the bench throw [23]; the prone bench pull [60,67]; the countermovement jump [61]; the power snatch, clean, and jerk [48]; and the hexagonal barbell deadlift. Validity and reliability studies of IMUs during Olympic lifts are quite limited [61], and thus it is believed that IMUs should be tested for different parts of the Olympic lifts.

In total, eight IMU models were used in the studies examined in this systematic review. Some studies were conducted using the BarSensei [27,46], the Gyko [49], the Myotest [56,58,59,65,67,69], the PUSH Band [23,24,48,54,64,66], the Wimu Real Track [6,60,62,68], the PASCO [61,70], and the Rehagait [63].

### 4.1. Validity of IMU for Estimation of Barbell Velocity

Twenty-one of the studies in this systematic review investigated validity (see Table 4). Specifically, IMU were compared with linear transducers in seven studies, a contact platform in six studies, the field computation method in one study, and a motion capture system in six studies (see Table 4 to a detail information). Among the included studies, nine tested the back squat; nine the bench press; one the hip thrust; one the bench throw; one the prone bench pull; two the countermovement jump; one the power snatch, clean, and jerk; and one the hexagonal barbell deadlift.

Overall, eight different IMU models were tested for validity. The reviewed studies were conducted using the BarSensei [27,46], the Gyko [49], the Myotest [56,58,59,65,67,69], the PUSH Band [23,24,48,54,64,66], the Wimu Real Track [6,60,62,68], the PASCO [61,70], and the Rehagait [63]. Four studies [27,46,60,67] reported a lack of validity of IMU equipment.

Validity studies also compared the different pieces of equipment with which IMUs were compared. The most detailed investigations are those tested with 3D camera measurement systems and force platforms, which are the gold standard. The variety of equipment used for validity comparisons can lead to differences. Differences in diversity between devices may be due to different sampling methods and the way raw data signals are processed in the software. Therefore, practitioners of IMUs should avoid using different devices interchangeably during the long-term monitoring of athletes.

The statistical methods and working designs of the equipment tested for validity differed. According to brands and methods for the BarSensei model used to test the validity of the findings of IMUs, the SEE values of validity were between 0.03 and 0.06 m•s^−1^ [46]. For the Gyko Sport model, the SEE and Pearson’s *r* values were 0.18 m•s^−1^ and 0.79, respectively [49]. For the Beast Sensor model, the SEE values ranged between 0.07 and 0.05 m•s^−1^, and Pearson’s *r* values ranged between 0.76 and 0.98 [24,34]. For the Myotest sensor model, the SEE values ranged between 0.01 and 26.6 m•s^−1^, Pearson’s *r* values ranged between 0.38 and 0.92, and the *R^2^* values ranged between 0.59 and 0.97 [56,58,59,65,67,69]. For the PUSH Band sensor model, the SEE values ranged between 0.135 and 0.091 m•s^−1^, Pearson’s *r* values ranged between 0.97 and 0.90, and the *R^2^* values were around 0.85 [6,23,24,48,64,66]. For the Wimu RealTrack Systems sensor model, the SEE values were around 0.030 m•s^−1^, Pearson’s *r* values ranged between 0.009 and 0.60, and the *R^2^* values ranged between 0.95 and 0.77 [28,60,68]. For the PASCO sensor model, Pearson’s *r* values ranged between 0.84 and 0.93 [61,70]. For the Barsensei model, the SEE values of validity were between 0.03 and 0.06 m•s^−1^ [46]. For the Gyko Sport model, the SEE values and Pearson’s *r* were 0.18 and 0.79, respecitvely [49]. For the Beast Sensor model, the SEE values were between 0.07 and 0.05 m•s^−1^ and Pearson’s *r* values were between 0.76 and 0.98 [24,34]. For the Myotest sensor model, the SEE values were between 0.01 and 26.6 m•s^−1^, Pearson’s *r* values were between 0.38 and 0.92, and *R^2^* values were between 0.59 and 0.97 [56,58,59,65,67,69]. For the PUSH Band sensor model, the SEE values were between 0.135 m and 0.091 m•s^−1^, Pearson’s *r* values were between 0.97 and 0.90, and *R^2^* value was 0.85 [6,23,24,48,64,66]. For the Wimu RealTrack Systems sensor model, the SEE value was 0.030 m•s^−1^, Pearson’s *r* values were between 0.009 and 0.60, and *R^2^* values were between 0.95 and 0.77 [28,60,68]. For the PASCO sensor model, Pearson’s *r* values were between 0.84 and 0.93 [61,70].

Considering the scenarios in which instruments may not be recommended, we found that the Wimu and Myoset may not be appropriate for measuring countermovement jumps, while Barsensei is not recommended for measuring velocity in squat and back squat exercises. The Myotest, Push Bando, Wimu, and Pasco revealed validity for measuring the main weight-room exercises such as bench press, bench throw, squat (front and back), or deadlift. The experience or type of competitive level of the participants had no effect on the tests.

In light of the findings revealed in the systematic review, sports scientists and practitioners should question the validity of the IMUs they use during exercises. Even if they do not have appropriate conditions to validate UMIs, it is recommended that they use validated equipment as shown by the data discussed in this systematic review.

### 4.2. Reliability of IMU for Estimation of Barbell Velocity

Twenty-one of the studies in this systematic review investigated reliability (see Table 5). Overall, seven different IMU models were tested for reliability. The studies were conducted using the BarSensei [27,46], the Gyko [49], the Myotest [56,57,58,65,67,69], the PUSH Band [23,24,48,64,66], the Wimu Real Track [6,60,62,68], and the PASCO [61]. Four studies [24,27,46,67] reported a lack of reliability in IMU equipment. The movements in which the equipment was tested in these studies reporting low reliability, the participant group, and biological differences should also be considered.

Reliability findings of IMUs, according to the brands and methods for the BarSensei model, the ICC values of validity were between 0.273 and 0.451, and the CV values ranged between 10% and 30% [27,46]. For the Gyko Sport model, the ICC value of reliability was 0.774 [49]. For the Beast Sensor model, the ICC values of reliability were between 0.36 and 0.99, and the CV values were around 35% [24,32]. For the Myotest model, the ICC values of reliability were between 0.35 and 0.97, the CV values were between 2.1% and 36.5%, and the SEM values of reliability were between 3% and 990 [56,57,58,65,67]. For the PUSH Band model, the ICC values of reliability were between 0.58 and 0.97, the CV values were between 4.2% and 13.7%, and the SEM values of the reliability were between 0.008 and 9.34 m•s^−1^ [23,24,48,64,66]. Depending on the number of studies, the PUSH BAND appears to be the IMU-based device that provides the most reliable data. For the Wimu RealTrack Systems model, the ICC values of reliability were between 0.81 and 0.97, the CV values were between 2.60% and 17%, and the SEM values of the reliability were between 0.007 and 0.11 m•s^−1^ [60,62,68]. For the PASCO model, the ICC values of reliability were between 0.95 and 0.99, and the SEM values of the reliability were between 0.55 and 1.77 m•s^−1^ [61].

In brief, the Myotest did not reveal enough levels of precision (reliability) for measuring countermovement jump, while Barsensei was not precise for measuring velocity in squat and back squat exercises. The Myotest, Push Bando, Wimu, and Pasco revealed precision for measuring the main weight-room exercises such as bench press, bench throw, squat (front and back), or deadlift. This is extremely important since instruments must be as must precise as possible in order to provide useful and sensitive information about readiness monitoring in sports, particularly for VBT.

The studies discussed in this systematic review investigated the reliability of the devices in certain movement patterns in field conditions. According to the authors, it is thought that studies on the long-term use of investigations of the reliability of IMUs should be designed to minimize the variables that might arise as a result of biological differences in longer use. However, it is also thought that malfunctions in the software data flow originating from the manufacturer may occur, and the disruptions in this data flow may affect the data reliability in IMUs. It is thought that research should be conducted to examine whether software and mobile phone applications that reflect instant data of IMU models transmit data reliably in real time. Among the studies discussed in this systematic review, none considered this situation. At the same time, to the best of the authors’ knowledge, there is no study in the literature that investigates the effects of software data flows on reliability.

### 4.3. Study Limitations, Future Research, and Practical Implications

Most of the research in this systematic review examined the validity and reliability of IMUs during movements performed in a single plane. This systematic review only tested the validity and reliability of the Flores et al. [61] Olympic lifts and IMUs. Practitioners, sports scientists, and strength and conditioning coaches often use multi-directional Olympic lifts as opposed to the limited movement patterns used in research. Future studies should examine the validity and reliability of IMUs during the Olympic lifts that appear as a dark zone.

The studies discussed in this systematic review generally consist of short-term research designs. Since IMUs are used by people with biological differences, it should be considered that long-term biological changes may affect the validity and reliability of IMUs in the long term. To the best of the authors’ knowledge, there are no studies examining the validity and reliability of long-term IMUs. For this reason, future studies should examine the validity and reliability of data to uncover insights about the long-term use of IMUs.

There may be some factors affecting the validity and reliability of IMUs beyond those that researchers have considered in their experimental designs. Some of these factors may be caused by the manufacturer. For example, it is believed that data transferred to software and mobile phone applications in real time result in errors in validity and reliability due to software malfunctions. In order for sports scientists, practitioners, and strength training coaches to use IMUs in a valid and reliable way, the effects of software factors on data quality should be investigated in future studies.

Table 6 presents a summary of the validity and reliability of different IMUs that may help coaches choose a model.

## 5. Conclusions

This present systematic review summarized evidence about the validity and reliability of IMUs for measuring barbell velocity. A total of eight models were tested across the 22 included articles. The Barsensei was not valid and reliable in the studies reports. The Gyko sport, Beast Sensor, and PASCO were valid and reliable in all reports. The Myotest, PUSH band, and Wimu RealTrack were valid and reliable in the majority of the reports. The Rehagait was valid. Therefore, from the eight included models, seven can be used with some evidence of being accurate and precise. This evidence provides important information for coaches who need accurate information about barbell velocity to control the external load imposed on athletes and to be sensitive to human variations without any meaningful bias generated by measurement instruments.

## Figures and Tables

**Table 1:** Eligibility criteria.

Inclusion Criteria	Exclusion Criteria
Test of a wearable wireless IMU.	Instruments other than wearable wireless IMU.
Tests were conducted in barbell movements.	The tests were not conducted in barbell movements (e.g., human movements, other instruments).
Estimation of barbell velocity (m/s).	Estimation of other outcomes than velocity (e.g., displacement).
In the case of validity, the IMU was compared with (i) an isoinertial dynamometer consisting in cable-extension linear position transducer, or (ii) optoelectronic system.	For validity, the IMU was compared with other instrument than isoinertial dynamometer or optoelectronic system (e.g., smartphone application; other IMU).
In the case of validity, one of the following measures were included: (i) typical error, (ii) mean absolute error, (iii) correlation coefficient, and (iv) standard error of the estimate.	For validity, outcomes presented are not typical error, mean absolute error, correlation coefficient, or standard error of estimate.
In the case of reliability, one of the following measures were included: (i) intraclass correlation test, (ii) coefficient of variation, (iii) standardized typical error and (iv) standard error of measurement.	For reliability, outcomes presented are not (i) intraclass correlation test, (ii) coefficient of variation, (iii) standardized typical error, and (iv) standard error of measurement.
Only original and full-text studies written in English.	Written in languages other than English. Article types other than original (e.g., reviews, letters to editors, trial registrations, proposals for protocols, editorials, book chapters, and conference abstracts).

**Table 2:** Methodological assessment of the included studies.

Reference	1	2	3	4	5	6	7	8	9	10	Quality
Abbott et al. [46]	1	1	0	1	1	1	1	1	1	1	High
Arede et al. [49]	1	1	1	1	1	1	1	1	1	1	High
Balsalobre-Fernández et al. [32]	1	0	1	1	1	1	1	0	0	0	Low
Bampouras et al. [56]	1	0	1	1	1	1	1	0	1	0	Low
Beckham et al. [27]	1	0	1	1	1	1	1	1	1	1	High
Caruso et al. [57]	1	1	1	1	1	1	1	0	0	0	Low
Comstock et al. [58]	1	1	1	1	1	1	1	1	1	1	High
Courel-Ibañez et al. [23]	1	0	1	1	1	1	1	1	1	0	High
Crewther et al. [59]	1	1	1	1	1	1	1	1	1	1	High
Ferro et al. [60]	1	1	1	1	1	1	0	1	1	0	High
Flores et al. [61]	1	1	0	1	1	1	1	0	1	1	High
García-Pinillos et al. [62]	1	0	1	1	1	1	1	1	1	1	High
García Mateo [63]	1	1	1	1	1	1	1	1	1	1	High
Jovanovic and Jukic [48]	1	1	1	1	1	1	1	0	0	1	High
Lake et al. [64]	1	1	1	1	1	1	1	1	1	0	High
Lorenzetti et al. [65]	1	0	0	1	1	1	1	1	0	1	Low
McGrath et al. [66]	1	0	0	1	1	1	1	0	1	0	Low
McMaster et al. [67]	1	0	1	1	1	1	1	1	1	0	High
Muyor et al. [68]	1	0	1	1	1	1	1	1	0	1	High
Pérez-Castilla et al. [24]	1	1	1	1	1	1	1	1	1	1	High
Rahmani et al. [69]	1	0	1	1	1	1	1	1	1	0	High
Sato et al. [70]	1	1	1	1	1	1	1	1	1	0	High

Note: Provide in the abstract an informative and balanced summary of what was done and what was found (item 1); state-specific objectives, including any prespecified hypotheses (item 2). Give the eligibility criteria, and the sources and methods of selection of participants (item 3); for each variable of interest, give sources of data and details of methods of assessment (measurement). Describe comparability of assessment methods if there is more than one group (item 4); explain how quantitative variables were handled in the analyses. If applicable, describe which groupings were chosen and why (item 5); give characteristics of study participants (item 6); summarize key results with reference to study objectives (item 7); discuss limitations of the study, considering sources of potential bias or imprecision. Discuss both direction and magnitude of any potential bias (item 8); give a cautious overall interpretation of results considering objectives, limitations, multiplicity of analyses, results from similar studies, and other relevant evidence (item 9); give the source of funding and the role of the funders for the present study and, if applicable, for the original study on which the present article is based (item 10).

**Table 3:** Study characteristics.

Study	IMU Brand and Model	IMU Characteristics	Comparator Characteristics	N/Sex/Population	Age (y)	Experimental Protocol	Movement	Validity Outcomes	Reliability Outcomes
Abbott et al. [46]	Barsensei, (Assess2Perform, Montrose, USA)	Triaxial IMU (100 Hz)	3DMOCAP, 4 cameras, Vicon System, United Kingdom (100Hz)	N = 16, men, resistance-trained males	25.9 ± 5.2	1RM squat test protocol, beginning at 20% of self-reported 1RM and progressing in 5–10% until failure	Squat	SEE	CV
Arede et al. [49]	Gyko Sport (Microgate, Bolzano, Italy)	Triaxial IMU (500 Hz)	SmartCoach Power Encoder linear transducer (100Hz)	N = 10, ND, basketball players	15.1 ± 1.0	Incremental test with repetitions at 40, 50, 60, 70, 80, and 90% of 1RM (six sets of two repetitions)	Bench press	SEE, Pearson’s *r*	ICC, Cronbach’s alpha
Balsalobre-Fernández et al. [32]	Beast sensor	Triaxial IMU (50 Hz)	SmartCoach Power Encoder linear transducer (1 kHz)	N = 10, men and women, competitive powerlifters	26.1 ± 3.9	1RM incremental test (five sets of repetitions with loads ranging ≈50–90% 1RM and one set of one repetition with 1RM)	Full squat, bench press, hip-thrust	SEE, Pearson’s *r*	ICC
Bampouras et al. [56]	Myotest (Sion, Switzerland)	Triaxial IMU (500 Hz)	Force platform	N = 30, men, physically active participants	28.3 ± 8.5	Two squat jumps were performed for each session in two different occasions interspaced by seven days	Squat jump with barbell	SEM, Pearson’s *r*	ICC, CV
Beckham et al. [27]	Barsensei, (Assess2Perform, Montrose, USA)	Triaxial IMU	GymAware Power Tool	N = 16, men and women, experienced participants	22.5 ± 2.6	Two sets of three repetitions at 45%, 60%, and 75% 1RM	Back squat	Mean difference	ICC
Caruso et al. [57]	Myotest (Sion, Switzerland)	Triaxial IMU (500 Hz)	-	N = 18, ND, American football players	ND	Three to six repetitions at 55, 65, 75, and 80% 1RM and additional 83% at 1RM	Front squat	-	ICC, CV, SEM
Comstock et al. [58]	Myotest (Myotest Inc, Switzerland)	Triaxial IMU (200 Hz)	Force platform	N = 97, men and women	24.2 ± 4.2	Three sets of repetitions at 30% and 1RM	Bench press, bench throw, squat	*R^2^*	ICC
Courel-Ibañez et al. [23]	PUSH Band (PUSH Inc., Toronto, Canada)	Triaxial IMU (200 Hz)	T-Force Dynamic Measurement System (1000 Hz)	N = 17, men	26.2 ± 3.6	Two sets of five repetitions, seven increasing loads (20-30-40-50-60-70- 80 kg)	Bench press, full squat, and prone bench pull	SEM, SEE, SDC, BIAS	ICC, CV, CCC, MSD
Crewther et al. [59]	Myotest (Myotest Inc, Switzerland)	Triaxial IMU (200 Hz)	Kistler portable force plate (Type 92866AA)	N = 12, men	28.8 ± 6.8	2 × single repetition were performed with 20, 40, 60, and 80 kg loads	Squats	Pearson’s *r*, systematic bias, random error	-
Ferro et al. [60]	Wimu RealTrack Systems (Almeria, Spain)	Triaxial IMU (1000 Hz)	Kistler Holding AG, Switzerland (1000 Hz), SmartCoach Power Encoder linear transducer	N = 9, men	20.78 ± 2.11	Five jumps were made in each of 6 series with a 20 kg barbell +0, +5, +10, +15, +20, and +25 kg	CMJ loaded	LoA, BIAS, CI, *R^2^*	ICC, TE, CV, SWC
Flores et al. [61]	PASCO (Roseville, California)	Triaxial IMU (100 Hz)	3DMOCAP, 4 cameras, Vicon System, United Kingdom (100Hz)	N = 11, men	27.47 ± 3.61	Subjects randomly performed three sets of one repetition with different loads, ranging from 30 to 90% of 1RM, using loads between 50 and 140 kg	Order of the exercises was power snatch, power clean, and jerk from the rack	Pearson’s *r*, ME, CV	ICC, SEM, ES
García-Pinillos et al. [62]	Wimu RealTrack Systems (Almeria, Spain)	Triaxial IMU (1000 Hz)	T-Force Dynamic Measurement System (1000 Hz)	N = 19, men	23.7 ± 2.8	The maximal test was applied by gradually adding 20 kg to the bar	Half-squat	Pearson’s *r*, LoA, systematic bias, random error, *R^2^*	CV, SEM
García Mateo [63]	RehaGait	Triaxial IMU	High-speed smartphone camera (MyLift)	N = 6, ND	25.6 ± 3.26	Fifteen repetitions of were performed with a bar less than 1 kg	Squats	Paired samples *t*-test	-
Jovanovic and Jukic [48]	PUSH Band (PUSH Inc., Toronto, Canada)	Triaxial IMU (200 Hz)	GymAware Power Tool (Kinetic Performance Technologies, Canberra, Australia)	N = 12, men	26.1 ± 4.3	1RM incremental test and sets to failure were performed with 90 and 80% of previously established 1RM	Hexagonal barbell deadlift	OLP regression, RSE, BIAS, Pearson’s *r*	SESOI, SDC
Lake et al. [64]	PUSH Band (PUSH Inc., Toronto, Canada)	Triaxial IMU (200 Hz)	3DMOCAP, 4 cameras, Vicon System, United Kingdom (100 Hz)	N = 14, men	22.1 ± 2.6	They performed three sets of three repetitions with 60% 1RM before progressing to perform three sets of one repetition with 90% 1RM	Bench press	BIAS, ordinary least products regression	Confidence limits, least products regression, ICC, CV
Lorenzetti et al. [65]	Myotest (Sion, Switzerland)	Triaxial IMU (200 Hz)	3DMOCAP, 16 cameras, Vicon System, United Kingdom (100 Hz)	N = 9, men	30.9 ± 5.9	Participants performed 2 × 5 traditional squats with a weight of 70% of their 1RM and 2 × 5 ballistic squats with a weight of 25 kg	Squat, ballistic squat	Pearson’s *r*	Root mean square error
McGrath et al. [66]	PUSH Band (PUSH Inc., Toronto, Canada)	Triaxial IMU (200 Hz)	Eagle motion capture system (Santa Rosa, California)	N = 10, ND	23.4 ± 6.8	One set of six repetitions at 40% 1RM, and one set of six repetitions at 80% 1RM	Bench press	Systematic bias and random error, *R^2^*	ICC
McMaster et al. [67]	Myotest (Sion, Switzerland)	Triaxial IMU (200 Hz)	Tri-axial force plate (Advanced Mechanical Technology, Inc., Acupower, Watertown, MA, USA)	N = 18, ND	21.6 ± 2.9	Weightless CMJ twice in a row	CMJ	Pearson’s *r*	ICC, SEM, ES
Muyor et al. [68]	Wimu RealTrack Systems (Almeria, Spain)	Triaxial IMU (1000 Hz)	T-Force Dynamic Measurement System	N = 23, men	22.3 ± 3.2	One set of 15 repetitions 10% RM, 10 repetitions 40% RM, 80% RM	Back squat	Systematic bias, effect size d, SEM, *R^2^*, SEE, ICC	Systematic bias, effect size d, SEM, ICC, CV
Pérez-Castilla et al. [24]	PUSH Band (PUSH Inc., Toronto, Canada), Beast Sensor (Beast Technologies Srl.)	Triaxial IMU	Trio-OptiTrack. Trio-OptiTrack (V120:Trio; OptiTrack, Natu- ralPoint, Inc.)	N = 14, men	22.9 ± 1.6	Three repetitions were executed, each with five relative loads of 45, 55, 65, 75, and 85% of 1RM	Bench press	Systematic bias, Pearson’s *r*	CV, ICC
Rahmani et al. [69]	Myotest (Sion, Switzerland)	Triaxial IMU (500 Hz)	Field computation method	N = 12, men	28.2 ± 9.8	10 reps at 17 kg, 8 at 27 kg, 6 at 37 kg, 4 at 47 kg, 3 at 57 kg, 2 at 67 kg	Bench press	SEE, *R^2^*	CV, ICC
Sato et al. [70]	PASCO (Roseville, California)	Triaxial IMU (100 Hz)	A high-speed video camera (HSV-400, NAC Image Technology, Japan)	N = 7, men	24.29 ± 2.98	Each participant made two trials with a weight of 40 kg	Barbell high-pull	Pearson’s *r*	-

RM: repetition maximum; CV: % of coefficient of variation; SEE: standard error of the estimate; SEM: standard error of measurement; ND: no defined; Pearson’s *r*: Pearson’s product moment correlation coefficient; ICC: intra-class correlation; y: years-old, SDC: smallest detectable change, CCC: Lin’s concordance correlation coefficient, MSD: mean square deviation, LoA: limits of agreement, SWC: smallest worthwhile change, ES: effect size, *R^2^*: Bland–Altman correlation coefficient, ME: method error, OLP: ordinary least products, RSE: residual standard error, SESOI: smallest effect size of interest; IMU: inertial measurement unit.

**Table 4:** Validity of IMU for estimation of barbell velocity.

Study	IMU Brand and Model	SEE	Correlation Coefficient	Evidence
Abbott et al. [46]	Barsensei, (Assess2Perform, Montrose, USA)	0.03 to 0.06 m•s^−1^	-	Not valid
Beckham et al. [27]	Barsensei, (Assess2Perform, Montrose, USA)	-	-	Not valid
Arede et al. [49]	Gyko Sport (Microgate, Bolzano, Italy)	0.18 m•s^−1^	*r* = 0.79	Valid
Balsalobre-Fernández et al. [34]	Beast Sensor	BW 0.04–0.07 m•s^−1^ | BB 0.04–0.05 m•s^−1^	*BW r* = 0.94–0.98 BB *r* = 0.97–0.98	Valid
Pérez-Castilla et al. [24]	Beast Sensor	-	*r = 0.76*	Valid
Bampouras et al. [56]	Myotest (Sion, Switzerland)	-	*r = 0.815*	Valid
Comstock et al. [58]	Myotest (Sion, Switzerland)	-	Bench press *R^2^* = 0.92, bench throw *R^2^* = 0.92, squat *R^2^* = 0.97	Valid
Crewther et al. [59]	Myotest (Sion, Switzerland)	-	*r = 0.92*	Valid
Lorenzetti et al. [65]	Myotest (Sion, Switzerland)	-	*r = 0.61*	Valid
McMaster et al. [67]	Myotest (Sion, Switzerland)	-	*A_hip_: 0.38* *A_bar_: 0.40*	Not valid
Rahmani et al. [69]	Myotest (Sion, Switzerland)	*F* (N): 30.2 $\bar{\nu }$ (m•s^−1^): 0.07 F0 (N): −18.7 v0 (m•s^−1^):−0.01 F-vslope (N/m•s^−1^): 6.1 Pmax (W): −26.6	*F* (N): *R^2^* = 0.95 $\bar{\nu }$ (m•s^−1^): *R^2^* = 0.89 F0 (N): *R^2^* = 0.93 v0 (m•s^−1^): *R^2^* = 0.59 F-vslope (N/m•s^−1^): *R^2^* = 0.99 Pmax (W): *R^2^* = 0.87	Valid
Courel-Ibañez et al. [23]	PUSH Band (PUSH Inc., Toronto, Canada)	Bench press: 0.135 m•s^−1^ Full squat: 0.091 m•s^−1^	Bench press: *r = 0.92* Squat: *r = 0.90*	Valid
Jovanovic and Jukic [48]	PUSH Band (PUSH Inc., Toronto, Canada)	-	*r = 0.915–0.948*	Valid
Lake et al. [64]	PUSH Band (PUSH Inc., Toronto, Canada)	-	*-*	Valid
McGrath et al. [66]	PUSH Band (PUSH Inc., Toronto, Canada)	-	*R^2^: 0.85*	Valid
Pérez-Castilla et al. [24]	PUSH Band (PUSH Inc., Toronto, Canada)	-	*r = 0.97*	Valid
Ferro et al. [60]	Wimu RealTrack Systems (Almeria, Spain)	-	*r = 0.009*	Not valid *
García-Pinillos et al. [28]	Wimu RealTrack Systems (Almeria, Spain)	-	*r = 0.60* *R^2^* = 0.77	Valid
Muyor et al. [68]	Wimu RealTrack Systems (Almeria, Spain)	0.030	*R^2^ = 0.95*	Valid
Flores et al. [61]	PASCO (Roseville, California)	-	*Power snatch r = 0.84* Power clean *r = 0.882* *Jerk r = 0.933*	Valid
Sato et al. [70]	PASCO (Roseville, California)	-	*r = 0.87*	Valid
García Mateo [63]	RehaGait	-	*-*	Valid

SEE: standard error of the estimate, BW: *Beast* Sensor wrist, BB: *Beast* Sensor barbell, *A_hip_:* accelerometer attached to the hip, *A_bar_*: accelerometer attached to the bar, F: mean force, $\bar{\nu }$: mean velocity, F0: maximal force at null velocity, v_0_: maximal velocity at null force, F-v_slope_: slope of the force–velocity relationship, Pmax: maximal power velocity relationship, N: newton, m•s^−1^: meter per second; * typical error = 0.09 m s^−1^.

**Table 5:** Reliability of IMU for estimation of barbell velocity.

Study	IMU Brand and Model	Intraclass Correlation Coefficient (ICC)	Coefficient of Variation (CV) (%)	Standard Error of Measurement (SEM)	Evidence
Abbott et al. [46]	Barsensei (Assess2Perform, Montrose, USA)	-	Between 10% and 30% in all intensities.	-	Not reliable
Beckham et al. [27]	Barsensei (Assess2Perform, Montrose, USA)	0.273–0.451	-	-	Not reliable
Arede et al. [49]	Gyko Sport (Microgate, Bolzano, Italy)	0.774	-	-	Reliable
Balsalobre-Fernández et al. [32]	Beast Sensor	BW 0.910–0.988 BB 0.922–0.990	-	-	Reliable
Pérez-Castilla et al. [24]	Beast Sensor	0.36	35.0%	-	Not reliable
Bampouras et al. [56]	Myotest (Sion, Switzerland)	F_ACC_ 0.90; P_ACC_ 0.80; V_ACC_ 0.84	F_ACC_ 2.1%, P_ACC_ 3.3% and V_ACC_ 3.2%	-	Reliable
Caruso et al. [57]	Myotest (Sion, Switzerland)	P55: 0.10 | P65: 0.86 | P75: 0.79 | P80–83: 0.97 | F55: 0.75 | F65: 0.85 | F75: 0.73 | F80–83: 0.81 |V55: 0.14 | V65: 0.89 | V75: 0.86 | V80–83:0.96	P55: 36.5 | P65: 20.4 | P75: 31.3 | P80-83: 17.8 | F55: 6.6 | F65: 4.7 | F75: 7.4 | F80–83: 7.8 | V55: 34.0 | V65: 20.5 | V75: 29.4 | V80–83: 21.0	P55: 990 | P65: 168.7 | P75: 379.9 | P80–83: 54.0 | F55: 50.0 | F65: 33.7 | F75: 75.6 | F80: 78.4 | V55: 106.0 | V65: 18.6 | V75: 26.4 | V80-83: 61	Reliable
Comstock et al. [58]	Myotest (Sion, Switzerland)	0.96	-	-	Reliable
Lorenzetti et al. [65]	Myotest (Sion, Switzerland)	-	-	-	Reliable
McMaster et al. [67]	Myotest (Sion, Switzerland)	*PF* *A_hip_: 0.80 | A_bar_: 0.83* PV and PP *A_hip_: 0.35 | A_bar_: 0.77*	-	*PF* *A_hip_: 3 | A_bar_: 13* PV and PP *A_hip_: 11 |A_bar_: 23*	Not reliable
Rahmani et al. [69]	Myotest (Sion, Switzerland)	0.90	<10%	-	Reliable
Courel-Ibañez et al. [23]	PUSH Band (PUSH Inc., Toronto, Canada)	MV full squat: 0.97 | PV full squat: 0.94 | MV bench press: 0.97 | PV bench press: 0.96	MV full squat: 5.6 | MV bench press: 12.2 | PV bench press: 13.7	MV bench press: 0.08 m•s^−1^| PV bench press: 0.18 m•s^−1^| MV full squat: 0.06 m•s^−1^ | PV full squat: 0.09 m•s^−1^	Reliable
Jovanovic and Jukic [48]	PUSH Band (PUSH Inc., Toronto, Canada)	-	-	-	Reliable
Lake et al. [64]	PUSH Band (PUSH Inc., Toronto, Canada)	PV 60% 1RM: 0.94 | MV 60% 1RM: 0.93 | PV 90% 1RM: 0.95 | MV 90% 1RM: 0.97	PV 60% 1RM: 4.2 | MV 60% 1RM: 5.8 | PV 90% 1RM: 4.7 | MV 90% 1RM: 7.2	-	Reliable
McGrath et al. [66]	PUSH Band (PUSH Inc., Toronto, Canada)	0.97	-	-	Reliable
Pérez-Castilla et al. [24]	PUSH Band (PUSH Inc., Toronto, Canada)	0.58	-	9.34	Not reliable
Ferro et al. [60]	Wimu RealTrack Systems (Almeria, Spain)	0.81	4.88	-	Reliable
García-Pinillos et al. [62]	Wimu RealTrack Systems (Almeria, Spain)	-	6–17	0.02–0.11 m•s^−1^	Reliable
Muyor et al. [68]	Wimu RealTrack Systems (Almeria, Spain)	40% concentric phase: 0.97 | 40% eccentric phase: 0.95 | 80% concentric phase: 0.90 | 80% eccentric phase: 0.92	40% concentric phase: 2.60 | 40% eccentric phase: 3.79 | 80% concentric phase: 3.53 | 80% eccentric phase: 4.51	40% concentric phase: 0.007 m•s^−1^| 40% eccentric phase: 0.013 m•s^−1^| 80% concentric phase: 0.011 m•s^−1^| 80% eccentric phase: 0.010 m•s^−1^	Reliable
Flores et al. [61]	PASCO (Roseville, California)	POWER SNATCH (up to pull phase): 0.95 | POWER CLEAN (up to pull phase): 0.96 | JERK (up to catch position): 0.99	-	POWER SNATCH (up to pull phase): 1.77 | POWER CLEAN (up to pull phase): 1 | JERK (up to catch position): 0.55	Reliable

BW: Beast Sensor wrist; BB: *Beast* Sensor barbell; F_ACC_: force from accelerometer; P_ACC_: power from accelerometer; V_ACC_: velocity from accelerometer; P: power; F: force; V: velocity; 55, 65, 75, and 80–83% 1RM (repetition maximum: MV: mean velocity; PV: peak velocity; A_hip_: accelerometer attached to the hip; A_bar_*:* accelerometer attached to the bar.

**Table 6:** Summary of validity and reliability of different IMU models.

	Barsensei (Assess2Perform, USA)	Gyko Sport (Microgate, Italy)	Beast Sensor	Myotest (Sion, Switzerland)	PUSH Band (PUSH Inc., Toronto, Canada)	Wimu RealTrack Systems, (Almeria, Spain)	PASCO (Rosevile, California)	RehaGait
Validity	Abbott et al. [46] Not valid Beckham et al. [27] Not Valid	Arede et al. [49] Valid	Balsalobre-Fernández et al. [34] Valid Pérez-Castilla et al. [28] Valid	Bampouras et al. [56] Valid Comstock et al. [58] Valid Crewther et al. [59] Valid Lorenzetti et al. [65] Valid McMaster et al. [67] Not Valid Rahmani et al. [69] Valid	Courel-Ibañez et al. [23] Valid Jovanovic and Jukic [48] Valid Lake et al. [64] Valid McGrath et al. [66] Valid Pérez-Castilla et al. [24] Valid	Ferro et al. [60] Not Valid García-Pinillos et al. [62] Valid Muyor et al. [68] Valid	Flores et al. [61] Valid Sato et al. [70] Valid	García Mateo [63] Valid
Reliability	Abbott et al. [46] Not Reliable Beckham et al. [27] Not Reliable	Arede et al. [49] Reliable (0.774)	Balsalobre-Fernández et al. [34] Reliable (0.910–988) Pérez-Castilla et al. [24] Not Reliable	Bampouras et al. [56] Reliable (0.80–0.90) Carusa et al. [57] Reliable (0.97–0.10) Comstock et al. [58] Reliable (0.96) Lorenzetti et al. [65] Reliable McMaster et al. [67] Not Reliable Rahmani et al. [69] Reliable (0.90)	Courel-Ibañez et al. [23] Reliable (0.94–0.97) Jovanovic and Jukic [48] Reliable Lake et al. [64] Reliable (0.93–0.97) McGrath et al. [66] Reliable (0.97) Pérez-Castilla et al. [24] Not Reliable	Ferro et al. [60] Reliable (0.81) García-Pinillos et al. [62] Reliable Muyor et al. [68] Reliable (0.92–0.95)	Flores et al. [61] Reliable	
